# Cartilage Oligomeric Matrix Protein, Diseases, and Therapeutic Opportunities

**DOI:** 10.3390/ijms23169253

**Published:** 2022-08-17

**Authors:** Jiarui Cui, Jiaming Zhang

**Affiliations:** 1 School of Rehabilitation and Health Preservation, Chengdu University of Traditional Chinese Medicine, Chengdu 610075, China; 2 Division of Bone and Mineral Research, Department of Oral Medicine, Infection and Immunity, Harvard School of Dental Medicine, Boston, MA 02115, USA

**Keywords:** cartilage oligomeric matrix protein, extracellular matrix, skeleton, malignancy, signaling

## Abstract

Cartilage oligomeric matrix protein (COMP) is an extracellular matrix (ECM) glycoprotein that is critical for collagen assembly and ECM stability. Mutations of COMP cause endoplasmic reticulum stress and chondrocyte apoptosis, resulting in rare skeleton diseases. The bouquet-like structure of COMP allows it to act as a bridging molecule that regulates cellular phenotype and function. COMP is able to interact with many other ECM components and binds directly to a variety of cellular receptors and growth factors. The roles of COMP in other skeleton diseases, such as osteoarthritis, have been implied. As a well-established biochemical marker, COMP indicates cartilage turnover associated with destruction. Recent exciting achievements indicate its involvement in other diseases, such as malignancy, cardiovascular diseases, and tissue fibrosis. Here, we review the basic concepts of COMP and summarize its novel functions in the regulation of signaling events. These findings renew our understanding that COMP has a notable function in cell behavior and disease progression as a signaling regulator. Interestingly, COMP shows distinct functions in different diseases. Targeting COMP in malignancy may withdraw its beneficial effects on the vascular system and induce or aggravate cardiovascular diseases. COMP supplementation is a promising treatment for OA and aortic aneurysms while it may induce tissue fibrosis or cancer metastasis.

## 1. Introduction

Articular cartilage consists of dense extracellular matrix (ECM) and sparsely distributed chondrocytes [1,2]. ECM is composed of water, collagen, proteoglycans, and small amounts of non-collagenous proteins [3]. It should be mentioned that the water in ECM is not free and can be attracted to the matrix by glycosaminoglycans (GAGs) with a high negative charge [4]. GAGs may play an important role in maintaining bone toughness via retaining bound water in the matrix [5], and the loss of its major subtype, chondroitin sulfate, is associated with age-related deterioration of bone toughness [6]. Cartilage oligomeric matrix protein (COMP) is a non-collagenous ECM glycoprotein that belongs to the family of thrombospondins (TSPs), also known as TSP-5, that is primarily found in human skeleton system (articular cartilage, meniscus, ligaments, tendons, and synovium) [7,8,9,10]. COMP is also expressed in the vitreous of the eye, heart, and vascular smooth muscle cells [11]. Moreover, COMP and its fragments can be present in body fluids. Serum and synovial COMP levels in the normal population are approximately 5.93 ± 1.95 µg/mL [12] and 33 ± 10 µg/mL [13], respectively. COMP is present in the earliest stages of human bone and joint development [14]. Strong staining for COMP was observed throughout the extracellular matrix of the cartilage in the human embryo at the 10th week of pregnancy [14]. 

COMP promotes the secretion and assembly of collagens and maintains the stability of ECM [14]. Mutations in the COMP gene can disrupt cartilage and bone formation, leading to rare skeleton diseases pseudoachondroplasia (PSACH) and multiple epiphyseal dysplasia type 1 (MED1) [15,16]. Moreover, accumulating evidence supports the links between COMP and other skeleton diseases, such as osteoarthritis (OA) and rheumatoid arthritis (RA). OA is characterized by notable articular cartilage degradation and other beyond-cartilage manifestations [17,18,19]. RA is a chronic autoimmune and inflammatory disease characterized by joint involvement with progressive cartilage and bone destruction [20]. COMP is considered to be a biochemical marker of cartilage turnover associated with destruction, and the indicated range appears to include, but is not limited to, articular cartilage and fibrocartilage (meniscus). Elevated serum COMP concentrations may also reflect higher levels of elastic cartilage (nasal, auricular, and tracheal cartilage) turnover during the active phase of relapsing polychondritis [21]. 

Due to the protective effects of COMP on cartilage, a thorough understanding of COMP functions and the related signaling pathways is beneficial for novel therapeutic strategies for cartilage degradation diseases. Recent exciting achievements have implied that COMP is an important gene contributing to other diseases, such as cancer/malignancy [22,23,24], cardiovascular diseases [25,26,27], and fibrosis [28,29,30]. These findings renewed our understanding of COMP functions and ECM biology beyond skeleton. The fact that COMP exhibits distinct functions in different diseases also challenges our previous conceptions about it. The non-selective effects of COMP supplementation may exert beneficial effects on certain diseases while inducing or exacerbating other diseases. In this review, we summarized the basic concepts of COMP, the evidence supporting COMP as a biomarker, and the novel mechanisms of COMP in skeleton and other diseases. 

## 2. Overview of COMP

**Figure 1:**
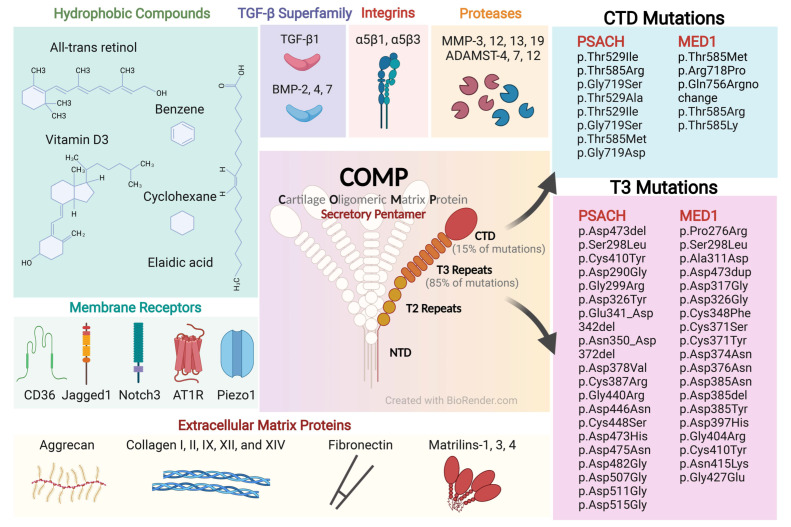
The structure, binding molecules, and mutations of COMP. COMP is a secretory extracellular matrix pentamer glycoprotein. Each monomer consists of an N-terminal coiled-coil domain (NTD), four type 2 (T2) epidermal growth factor-like repeats, seven type 3 (T3) calcium-binding repeats, and a C-terminal globular domain (CTD), assembling to a pentamer via its NTD. Due to its bouquet-like structure of pentamer, COMP interacts with many other ECM components and binds to a variety of cellular receptors and growth factors, acting as a bridging molecule that regulates cell phenotypes and functions. Mutations of COMP lead to genetic rare diseases pseudoachondroplasia (PSACH) and multiple epiphyseal dysplasia type 1 (MED1).

COMP is a secretory pentamer multidomain protein that belongs to TSP family subgroup B [31]. Remarkably, COMP is structurally conserved across mammalian species. Each monomer arm consists of an N-terminal coiled-coil domain (NTD), four type 2 epidermal growth factor (EGF)-like repeats, seven type 3 (T3) calcium-binding repeats, and a C-terminal globular domain (CTD), assembling to a pentamer via its NTD [32] (Figure 1). The T3 repeats have five highly conserved aspartic acid residues and two calcium-binding sites in each motif; the CTD is a lectin-like β-sandwich domain with 15 antiparallel β-strands and four calcium-binding sites [33]. The T3 repeats and CTD are of great interest because the majority of approximately 100 disease-causing mutations reported occurs within these regions (T3 repeats: ~85%; CTD: ~15%) [7] (Figure 1). Functionally, COMP is able to interact with many other cartilage ECM components, including type I, type II, type IX, type XII, and type XIV collagen, fibronectin, and proteoglycans [34,35,36,37,38]. Due to its unique bouquet-like arrangement of the five monomers that forms a cavity, COMP also interacts with growth factors and other hydrophobic compounds, such as Vitamin D3 and all-*trans* retinol [31], which make it a bridging molecule that regulates cell phenotypes and functions such as proliferation, differentiation, and attachment [39] (Figure 1). 

## 3. COMP and Skeleton Diseases

### 3.1. COMP and Cartilage Homeostasis

**Figure 2:**
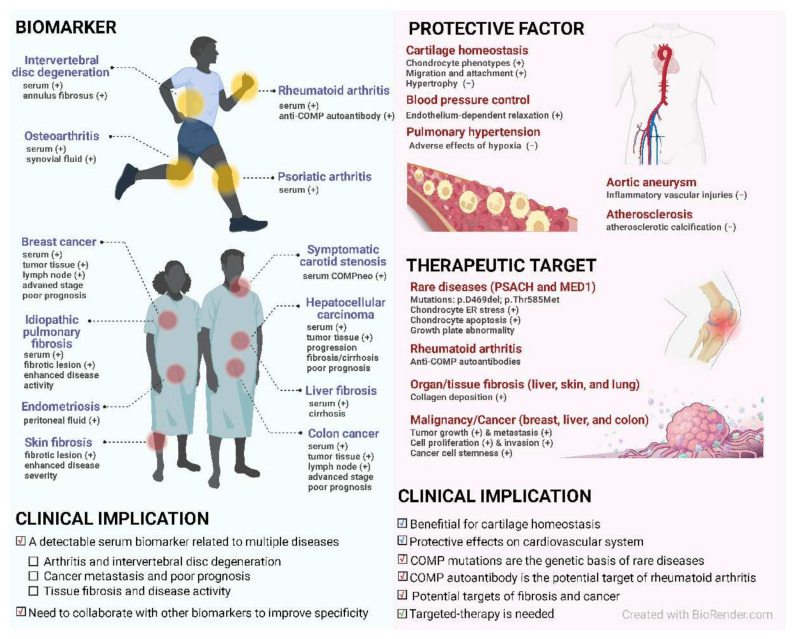
The clinical implications of COMP. COMP is widely associated with multiple diseases. COMP can act as a biomarker for osteoarthritis, rheumatoid arthritis, intervertebral disc degeneration, and psoriatic arthritis. Increased COMP in serum or synovial fluid indicates cartilage destruction. Moreover, serum COMP can be an indicator of cancer progression/poor prognosis, organ/tissue fibrosis, and other diseases/conditions. COMP shows distinct functions in different diseases. COMP is a protective factor for cartilage homeostasis, blood pressure control, pulmonary hypertension, and other cardiovascular diseases, while COMP is a potential target for rare skeleton diseases, rheumatoid arthritis, fibrosis, and cancer. Plus signs in parentheses in the blue panel indicate the increased levels of COMP in patients compared with healthy individuals or controls. Plus signs or minus signs in parentheses in the red panel indicate promotion or inhibition, respectively. PSACH, pseudoachondroplasia; MED1, multiple epiphyseal dysplasia type 1; COMPneo, COMP neoepitope, a fragment generated by COMP degradation; ER, endoplasmic reticulum.

**Figure 3:**
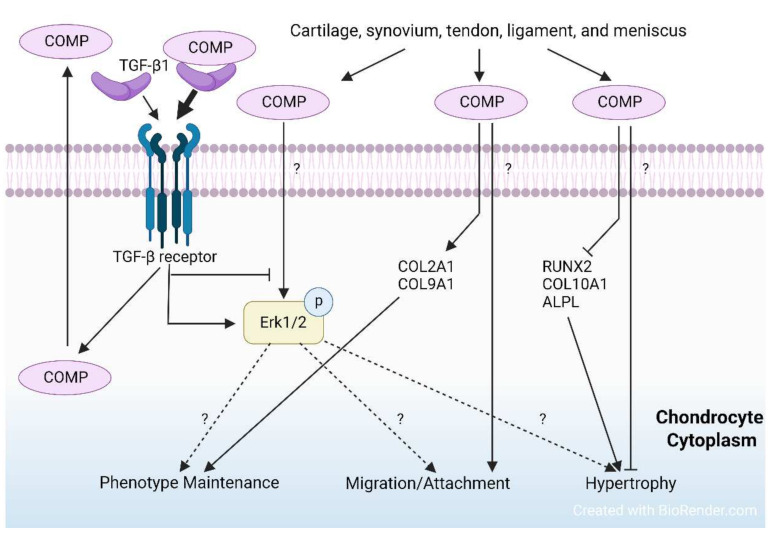
The functions and related signaling events of COMP in chondrocyte phenotype maintenance, migration, and attachment. In joint, COMP can be expressed in cartilage, synovium, tendon, ligament, and meniscus. COMP maintains chondrocyte phenotypes and promotes cell migration and attachment, while suppressing hypertrophy. COMP modifies the MAPK and TGF signaling pathways. COMP can induce the phosphorylation of Erk1/2, while this effect can be attenuated by TGF-β1. TGF-β1 can induce COMP expression and the interaction between COMP and TGF-β1 enhances the effects of TGF β signaling pathway, forming a regulation loop. Dotted lines and question marks indicate undetermined mechanisms.

The favorable effects of COMP on chondrocytes and cartilage have been implied by previous studies (Figure 2). Apart from its positive impacts on ECM organization by enhancing collagen secretion and fibrillogenesis [35,40], COMP binds directly to a variety of matrix proteins, cellular receptors, and growth factors, such as transforming growth factor-beta (TGF-β) superfamily (BMP-2, BMP-4, BMP-7, and TGF-β1) [7,41]. The binding of TGF-β1 and COMP enhances TGF-β1-induced intracellular signaling pathway in chondrocytes [41] (Figure 3). This suggests that its binding functions have a critical role in modifying cell signaling and maintaining cartilage homeostasis [1]. Moreover, COMP supplementation can delay chondrocyte hypertrophy by reducing the gene expression of Runx2, Col10a1, and Alpl [42] (Figure 3). 

### 3.2. COMP Mutations and Skeleton Diseases

The COMP mutations can significantly affect cartilage and bone growth, causing two types of autosomal dominant skeletal dysplasia disorders, PSACH and MED1 [16] (Figure 1 and Figure 2). These two diseases are separate but overlapping osteochondrodysplasias with similar clinical and pathological manifestations as well as genetic basis [43,44]. PSACH is characterized by dwarfed-like short stature with short limbs, loose joints, and early-onset osteoarthropathy. MED1 is a relatively mild chondrodysplasia but shows similar clinical manifestations [43]. 

The first COMP mutations were identified in 1995 in both PSACH and MED1 patients [16,45]. The majority of the COMP mutations discovered by subsequent studies were shown to occur mainly in the T3 calcium-binding domain. The deletion of aspartic acid residue 469, often known as D469del, is the most common COMP mutation, accounting for roughly 30% of PSACH cases [46,47]. Basic studies on the D469del-COMP mice have built a strong causal link between this mutation and PSACH [48,49]. At one month of age, the D469del-COMP mice have typical dwarfed appearances and PSACH chondrocyte pathology, including abnormal growth plate structure, massively-enlarged rough endoplasmic reticulum (rER) in chondrocytes, intracellular assembled ECM proteins such as type II and type IX collagen and matrilin-3, and increased chondrocyte apoptosis [48]. These phenotypes are the recapitulations of the finding in human PSACH growth plate. Mechanistically, the mutations in the T3 region compromise calcium binding and protein folding, leading to the retention of COMP in the chondrocyte rER. This accumulation causes abnormal chondrocytes in a state of inflammation and oxidative stress via CHOP signaling, eventually resulting in early death of these cells and impairment of linear growth in long bones [15,50,51,52]. Moreover, persistent ER stress, inflammation, autophagy blockage, catabolism, and senescence are important for early-onset joint degeneration after COMP mutation [53]. Unbalanced adipogenesis and osteogenesis and delayed ossification may contribute to compromised bone mineral density, bone quality, and subchondral bone thickness, partially in a miR-223-dependent mechanism [15]. Compared with most mutations in the T3 repeat region, mutations in the CTD region, such as the single nucleotide substitution mutation p.Thr585Met (equivalent to p.Thr583Met in mice) [54], induce an unfolded protein and cell stress response that lead to significantly reduced chondrocyte proliferation and increased apoptosis without the retention of the mutant COMP in rER [55]. 

MED1 has a more diverse range of COMP mutations than PSACH [56]. In addition to missense mutations at conservative residues (p.Pro276Arg, p.Ser298Leu, p.Ala311Asp), a wide range of in-frame deletions, duplications, and deletions/insertions (p.Asp473dup) is covered [56,57]. According to the premise that the deletion of the p.Asp473del always resulted in PSACH whereas the insertion of the p.Asp473dup always resulted in MED, the insertion of aspartic acid into the C-type motif of T36 may be less detrimental to the protein fold and structure than its deletion [56]. 

### 3.3. The Use of COMP as a Biomarker for OA, RA, and Other Skeleton Diseases

The reliable diagnosis standards of early OA are rare while the existing standards for diagnosing OA based on clinical symptoms and radiographic criteria have clinical achievements in late OA. The diagnostic criteria for early knee OA in the CHECK study have a fair predictive ability in individuals presenting with knee pain [58], while more validations are required. In fact, irreversible disease progression and joint degradation have already occurred when physical or radiographic evidence is established, delaying the optimal time for early treatment [59]. Moreover, the OA clinical symptoms, such as pain, are commonly delayed because the patients are often elderly and with pain tolerance, resulting in delayed diagnosis and treatment. Identification of reliable biochemical markers for the early diagnosis of OA and the prediction of disease progression is a primary priority in OA management [60]. It is important for the individuals with OA risk, such as trauma, who may have knee pain while no clear physical or radiographic evidence of OA is observed. It also helps to identify the endotypes among OA patients and monitor the effects of a treatment at an individual level [61]. 

**Figure 4:**
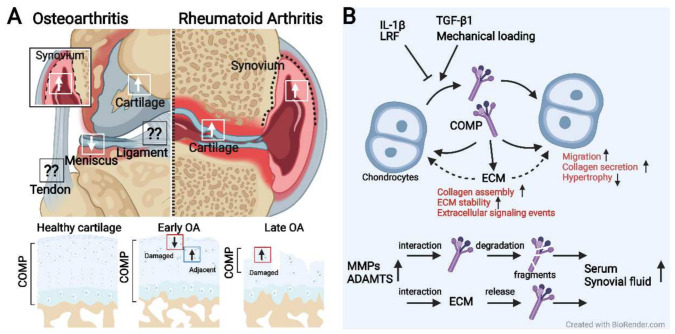
The COMP content, regulation, and catabolism in arthritic joints. (**A**) COMP content is increased in the cartilage and synovium of arthritic joints, while COMP is decreased in the meniscus of late osteoarthritis (OA). COMP content is ubiquitously and uniformly distributed throughout all layers in healthy cartilage. In the early OA, COMP content is decreased in the damaged region while increased in the adjacent region. In the late OA with extensive cartilage destruction, the localization of COMP is limited within the superficial region; pronounced intracellular staining of COMP can be detected in this region. (**B**) COMP expression is regulated by mechanical loading, TGF-β1, IL-1β, and transcriptional repressor LRF. The combined regulation of multiple factors may be the mechanism of the dynamic expression of COMP in OA. Secreted COMP interacts with extracellular matrix (ECM) components that influence cell signaling events and functions as ECM feedback to chondrocytes. COMP content in ECM can be degraded by matrix-degrading proteinases, leading to the release and fragmentation of COMP and the increased flow into serum and synovial fluid. MMPs, matrix metalloproteinases; ADAMTS, a family of multidomain extracellular protease enzymes. Up or down arrows indicate the increase or decrease of COMP content. Question mark indicates undetermined.

COMP may be a candidate biomarker for early OA (Figure 2). It is a well-established biochemical marker of cartilage turnover associated with cartilage destruction [62,63]. In chondrocytes and synovial cells, COMP expression can be activated by proinflammatory cytokines [64]. Mechanistically, COMP is released into the joint fluid following injury and during the early stages of OA [65] (Figure 4). Due to its involvements in cartilage degradation and inflammation, the clinical value of COMP has been implied and assessed in a certain number of studies [1,66]. However, the findings of COMP serving as a reliable biochemical marker are in conflict, which gave rise to the first meta-analysis (2019) that systematically reviewed the 35 human studies completed before January 2018 [59]. This study indicates a moderate performance of COMP in distinguishing between knee or hip OA patients and control subjects and predicting OA progression, while study size and diagnostic criteria did not significantly influence the performance. Moreover, subgroup analysis also showed that the performance of COMP in synovial fluid was better than the one in serum while serum COMP was more efficient in males than females [59]. Although this meta-analysis indicates that the performance of COMP in synovial fluid is preferable, it may be difficult to obtain synovial fluid in early OA. Importantly, serum COMP seems to be a more useful predictor, as a high level of serum COMP is significantly associated with early OA [67]. Still, more studies are required to standardize the criteria of COMP in clinical practice. 

COMP is a potential biomarker for the diagnosis and prognosis of RA [12,68,69,70,71] (Figure 2). Increasing serum-COMP levels in patients with early RA at first 3 months after diagnosis indicates an activated destructive process and significant joint damage progression in next 5 years [72]. More importantly, accumulating evidence indicates that COMP and its autoantibodies play pathogenic roles in experimental arthritis [73,74,75,76]. They may have an important role in RA due to its clinical relevance that COMP autoantibodies were detectable in synovium and serum from RA patients [77,78]. Moreover, serum COMP has recently been discovered to be a valid indicator for the diagnosis and prognosis of experimental intervertebral disc degeneration (IVDD) [79].

### 3.4. Treatments and Targeting COMP in OA and Other Pathologies

In OA, COMP expression is upregulated in chondrocytes adjacent to damaged cartilage [14], which may be related to repairing damage or supplementing matrix [14] (Figure 4). COMP content in the ECM decreases in early OA probably due to the cleavage of MMP13 [31,80], while COMP is re-expressed in late OA [14] (Figure 4). The re-expression may be due to the attempt of COMP to attract chondrocytes from adjacent locations to repopulate the defect [1]. OA cartilage in the late stages is damaged with a notable loss of ECM and cell component. The microenvironment of surviving chondrocytes and the cellular response to mechanical stress are changed. Moreover, without the ECM protection from excessive mechanical loading, chondrocytes in the late stages are assumed to be subjected to more strong mechanical stress than in the early stages. Mechanical loading is a stimulator of COMP expression, as the areas bearing the high load in human OA tissue in vivo show high COMP expression [14]. Unlike mechanical loading and TGF-β1 which induce COMP synthesis [1,81,82], IL-1β can suppress COMP expression in human OA chondrocytes, along with increased catabolic and reduced anabolic markers [83], indicating that COMP is under the regulation of IL-1β (Figure 4). Moreover, COMP is regulated by HDAC3 [84]. HDAC3 is increased by IL-1 in OA chondrocytes, while HDAC3 inhibition promotes histone H3 acetylation in the COMP promoter thus increasing COMP expression [84]. The interaction of COMP with other proteins requires a specific condition. For instance, a slight acidic condition (pH: 6.75) promotes the binding between COMP and TGF-β1 [41]. This suggests that the interaction may be changed as a result of the acidification of the OA matrix. A recent study (2021) showed that COMP alone can modulate collagen/proteoglycan synthesis to maintain chondrocyte phenotypes and facilitate chondrocyte migration and attachment while it has no independent effect on cell proliferation [1]. More importantly, this study found that COMP induces the phosphorylation of Erk1/2 in chondrocytes while this effect can be suppressed by TGF-β1 [1] (Figure 3). Interestingly, other ECM components, such as collagen II, have been found to inhibit the hypertrophy of articular chondrocytes via the Erk1/2 signaling pathway [85]. In OA, for currently unknown reasons, chondrocytes tend to switch from anabolic to catabolic Erk1/2 signals, leading to unstable chondrocyte phenotype and ECM degradation [1,86,87]. Although this study [1] did not involve OA model, the regulation of TGF-β1/COMP/ Erk1/2 would be of great interest because both signaling pathways are critical for cartilage degradation and repairment. Besides cartilage and synovium, COMP content in the other joint elements, such as tendon, ligament, and meniscus, remains unclear while a study showed that COMP is decreased in human meniscus and is significantly associated with OA development [9]. The understanding of COMP and its related signaling pathways is important to reveal the ECM-chondrocyte interaction in OA while a majority of studies focused on its potential as a biomarker and the detailed mechanism remains elusive. There are several points that may be important: (1) the influence on cartilage mechanical integrity and how this influence contributes to OA; (2) the effects on OA model in vitro and in vivo and the mechanisms by which COMP regulates OA chondrocyte; (3) the effects on other joint components in OA, such as tendon, ligament, meniscus, and synovium. 

C57BL/6NJ mice immunized with human COMP can develop a severe RA model that relies on COMP specific peptides [88]. The murine monoclonal anti-COMP antibody 15A11 induces RA in mice; its serum level is detectable at significantly higher levels in RA patients compared to healthy controls and positively correlated with disease activity. This study suggests that anti-COMP autoantibodies may play a pathogenic role in a subset of RA patients [78]. Moreover, COMP is expressed in both annulus fibrosus and nucleus pulposus of the lumbar spine and tail intervertebral discs in rats [89]. In human TNFα overexpressing transgenic (hTNFα-TG) mice, COMP is reduced in caudal annulus fibrosus (AF) along with robust cell death and immune cell infiltration, suggesting matrix destabilization and inflammation [90]. However, quantitative proteomic analysis revealed an elevated COMP level in AF of IVDD patients [91]. COMP mRNA and protein expression levels are much lower in the chondrocytes from Kaschin–Beck disease (KBD) patients [92]. When COMP is overexpressed, Survivin and SOX9 mRNA expression levels in KBD chondrocytes are significantly higher than in the control group, suggesting that COMP may play a role in the excessive KBD chondrocyte apoptosis by reducing Survivin and SOX9 expression [92].

## 4. COMP and Cancer/Cancer Malignancy/Cancer Progression

**Figure 5:**
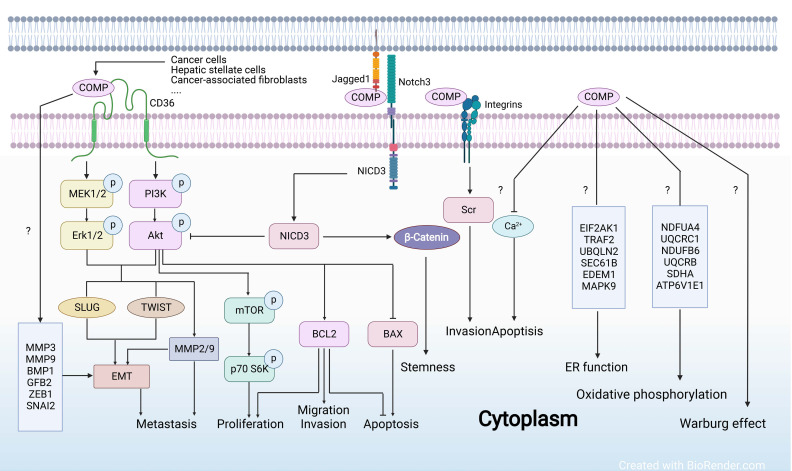
The functions and related signaling events of COMP in cancer cell behaviors and metabolisms. COMP binds to CD36, facilitating the activation of MEK/ERK and PI3K/Akt signaling pathways to promote proliferation, migration, invasion, and metastasis while suppressing apoptosis. COMP increases the interaction between Notch3 and its ligand Jagged1, thereby enhancing the activation of Jagged1-Notch3 signaling and cross-reactivity with PI3K/Akt and β-catenin, resulting in enhanced cancer stemness. COMP interacts with integrins to activate Scr, facilitating cancer cell invasion. COMP inhibits Ca^2+^ signaling, thereby promoting an anti-apoptotic effect. COMP modifies endoplasmic reticulum and mitochondrial functions to reduce endoplasmic reticulum stress and enhance the Warburg effect. Question marks indicate undetermined mechanisms.

COMP is also found to be highly expressed in tumor tissues, such as hepatocellular carcinoma [93], colon cancer [23], and breast cancer [94], and is often related to a high recurrence rate and low survival rate in cancer patients, serving as an independent prognostic biomarker (Figure 2) [94]. Moreover, accumulating evidence has indicated the potential of COMP as a therapeutic target (Figure 2 and Figure 5). Targeting COMP or its interaction with other proteins may be a promising approach for preventing tumor progression [23]. 

### 4.1. Hepatocellular Carcinoma

Elevated serum COMP is strongly associated with hepatocellular carcinoma (HCC) progression [95], suggesting that it can be used for non-invasive assessments of HCC progression. Moreover, the combination of COMP and GP73 showed a pronounced performance in detecting severe fibrosis/cirrhosis and predicting the development of HCC in patients with chronic liver diseases [30]. A recent study also showed that a multi-marker panel (AFP and PIVKA-II, with either IGFBP3, COMP or MMP3, plus age and sex) is efficient for the detection of early- and all-stage HCC [96]. COMP expression in HCC tissues is higher than in normal controls [93]. COMP driven from activated hepatic stellate cells (HSC) regulates mesenchymal gene expression and MMPs in HCC cells via CD36, a classic membrane receptor of the TSP family, and leads to abnormal phosphorylation of ERK and Akt [97] (Figure 5). The MEK/ERK and PI3K/Akt signaling pathways have been implicated in the regulation of tumor cell growth, metabolism, proliferation, and metastasis [22,98]. As a result, the pro-proliferative and pro-invasive effects of COMP are confirmed in HCC [97]. The application of Resolvin D1 to impair cancer-associated fibroblasts (CAFs)-derived COMP by targeting FPR2/ROS/FOXM1 signaling, thereby inhibiting the promotion of CAFs on the growth and metastasis of HCC tumors, has demonstrated that targeted matrix-derived COMP may be an effective strategy for blocking tumor-matrix interaction [99].

### 4.2. Colon Cancer

COMP expression levels in cancer tissues are significantly higher than in neighboring normal tissues [100]. Increased COMP expression in tumor tissue is related to advanced tumor node metastasis (TNM) stage and poor prognosis in stage III and IV patients [100]. Moreover, increased COMP expression is associated with consensus molecular subtype 4 (CMS4) but not tumor location and KRAS mutant status [101]. Wusterbarth et al. found that tumor COMP may be superior to Carcinoembryonic Antigen (CEA) as a prognosis biomarker [101]. Preoperative patients had much higher serum COMP levels than healthy donors, whereas serum COMP levels were significantly reduced after colectomy [100], indicating its potential in monitoring tumor burden. Recent studies have partially revealed its mechanisms related to poor clinical outcomes. COMP promotes the proliferation of CC cells by activating the PI3K/Akt/mTOR/p70S6K pathway [100] (Figure 5), suggesting that it could be a viable therapeutic target for CC. COMP is also substantially expressed in highly malignant colorectal cancer [23]. COMP knockdown prevents the metastasis and invasion of colorectal cancer, while COMP overexpression enhances epithelial-mesenchymal transition (EMT) [23]. Mechanistically, COMP interacts with the actin-binding protein Transgelin to regulate cytoskeletal remodeling and enhance the malignant progression of colorectal cancer [23]. Targeting the COMP-Transgelin interaction may be a promising strategy for inhibiting EMT and metastasis. Indeed, chrysin (a small flavonoid molecule) has been implied to target the COMP-Transgelin complex, exhibiting a prevention on EMT and malignant progression [23]. 

### 4.3. Breast Cancer

Serum COMP is elevated in the advanced breast cancer patients with metastasis and significantly associated with histological subtype, estrogen receptor positivity, and metastasis at diagnosis [102]. Moreover, elevated serum COMP could be an independent prognostic biomarker of survival in metastatic patients [102]. Tumor COMP expression is also an emerging independent prognosis indicator that is correlated with clinical parameters, including the number of lymph node metastases, estrogen and progesterone receptor positivity, Ki67 status, and poor survival and recurrence [94]. High COMP/BRD2 or COMP/BRD3 is correlated with poor prognosis, specifically decreased distant metastasis-free survival [103]. Furthermore, high COMP expression in lymph node metastases indicates reduced survival [24]. Previous studies have found that COMP can change the biological behaviors and functions of breast cancer cells. High COMP expression renders cancer cells resistant to apoptosis induction and endoplasmic reticulum stress and enhances the Warburg effect (Figure 5), which may be attributed to the prevention of Ca^2+^ release from ER [94,104]. COMP-expressing MDA-MB-231 cells form larger tumors than the controls [94]. Multiple lines of evidence strongly indicate the role of COMP in breast cancer metastasis. The most intense expression of COMP observed at the invasive margin and the retained strong COMP expression in node metastases strongly indicates that COMP enhances metastasis in vivo. Mechanistically, COMP enhances invasiveness by increasing MMP9 expression, while COMP has no effects on adhesion and migration of cancer cells in vitro [94]. It seems that COMP can promote cancer cell stemness. COMP increases the interaction between Notch3 and its ligand Jagged1, causing higher activation of Jagged1-Notch3 signaling and cross-reactivity with other important cancer-related pathways, such as AKT and β-catenin, resulting in the generation of a large number of cancer stem cells [105] (Figure 5). Interestingly, COMP is more abundant in exosomes from type 2 diabetes-derived or insulin-resistant adipocytes compared with in nondiabetic donor-derived adipocytes [103]. Exosome-derived COMP partially contributes to EMT in recipient cells by increasing MMP9, MMP3, BMP1, TGFB2, ZEB1, and SNAI2 (ER^+^ human breast cancer cell line, MCF7 cells) [103] (Figure 5). Beyond a prognostic biomarker, increased COMP (free in serum or derived by exosome) in circulation may target remote tissues and organs, including cancer, to modify their functions and make them to be more “stem cell-like” or “activated”.

### 4.4. Others

In prostate cancer, tumor COMP expression is associated with invasion and disease progression [104]. COMP-mediated invasion may be dependent on integrin/Scr signaling [104] (Figure 5). COMP modulates cell metabolism by inhibiting Ca^2+^ signaling, thus promoting an anti-apoptotic effect (Figure 5). Liu et al. reported that long non-coding RNA SNHG25 promotes the proliferation, invasion, and metastasis of epithelial ovarian cancer possibly through regulating COMP expression [106]. Wang et al. proposed that ADAMTS7 inhibits osteosarcoma progression by degrading COMP [107]. In lung adenocarcinoma, the co-mutation of EGFR^L858R^/TP53 is associated with poor prognosis and elevated COMP and ITGB8 expression levels [108]. COMP expression is upregulated in papillary thyroid carcinoma (PTC) tissue, which promotes the behaviors of PTC cells by activating the PI3K/Akt/Bcl-2 pathway [109] (Figure 5). Moreover, tumor COMP expression is positively correlated with tumor size, lymph node metastasis, and advanced TNM stage [110]. In gastric cancer (GC), tumor COMP has also been identified as a diagnostic and prognostic biomarker [111]. Moreover, COMP expression is associated with advanced pathological grade, immune cell infiltration, tumor mutation burden, and microsatellite instability [111]. Thiotepa, Idarubicin, and Triethylenemelamine were predicted to be the possible drugs targeting COMP in GC [111]. 

## 5. COMP and Cardiovascular Disease

**Figure 6:**
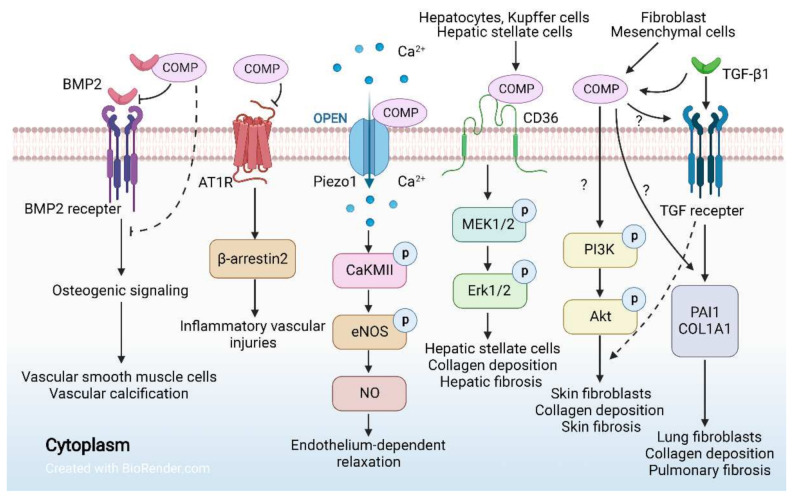
The functions and related signaling events of COMP in cardiovascular diseases and organ or tissue fibrosis. COMP has a favorable effect on vascular smooth muscle cells against calcification, inflammation, and hypertension. COMP binds to BMP2, thus blocking BMP signaling to suppress vascular calcification. COMP binds to AT1R to inhibit AngII-induced AT1R-β-arrestin2 signaling, thereby counteracting inflammatory vascular injuries. COMP interacts with Piezo1 to facilitate intracellular Ca^2+^ influx, thus increasing endothelial nitric oxide synthase activity and nitric oxide generation via CaKMII/eNO signaling. COMP promotes fibrosis by CD36/MEK/ERK, TGF-β, and PI3K/Akt signaling pathways. Moreover, COMP promotes TGF-β1 induced fibrosis in lung fibroblasts. Dotted lines and question marks indicate undetermined mechanisms.

COMP is a protective factor for cardiovascular system (Figure 2). Patients with pulmonary hypertension (PH), especially females, have lower serum COMP concentrations [25]. Serum COMP was shown to be negatively correlated to tricuspid annular plane systolic excursion and mean right atrial pressure [25]. The wall of pulmonary small arteries in COMP^−/−^mice was thicker than that of wild-type (WT) mice, while there is no difference in pulmonary hemodynamics between them [112]. Hypoxia-induced COMP deficiency leads to enhanced proliferation, increased oxidative stress, and decreased contractile phenotype markers of pulmonary artery smooth muscle cells [112,113]. COMP supplementation can reverse the adverse effects of hypoxia, implying that COMP is implicated in the pathogenesis of PH and can play a protective role [113]. Moreover, a recent study has revealed the role of COMP in blood pressure control [26]. Compared with WT mice, COMP^−/−^ mice display elevated blood pressure and impaired endothelium-dependent relaxation induced by acetylcholine [26]. Mechanistically, COMP directly interacts with Piezo1 and activates its downstream events by intracellular Ca^2+^ influx, thus increasing endothelial nitric oxide synthase activity and nitric oxide generation [26] (Figure 6). 

COMP is identified as an endogenous allosteric deviation modulator of angiotensin II type 1 (AT1) receptor. COMP deficiency activates the AT1 receptor/β-arrestin-2 signal (Figure 6), which exacerbates the activation of AT1 receptor-related diseases, such as abdominal aortic aneurysm [27]. Aortic aneurysm in COMP^−/−^ mice could be rescued by the application of a peptidomimetic that mimics the AT1-binding motif of COMP [27]. Human studies also show that COMP downregulation is associated with aortic aneurysm [114]. These findings suggest that COMP supplementation or mimic could be a novel therapeutic strategy for aortic aneurysm. 

COMP deficiency causes aging-related vascular dysfunction, implying that COMP is also critical in preventing vascular aging and the senescence of vascular smooth muscle cells (VSMC) [115]. COMP is responsible for maintaining the contractile phenotypes of VSMCs. Its deficiency promotes VSMC migration while exacerbating VSMC calcification and atherosclerosis [116]. Animal studies have shown that bone marrow transplantation of ApoE^−/−^COMP^−/−^ mice to ApoE^−/−^ mice increases the formation of atherosclerotic plaques, suggesting that bone marrow-derived COMP may play a crucial role in preventing atherosclerosis [117]. Mechanistically, COMP-deficient macrophages exert a phenotype shift characterized by atherogenic and osteogenic characters that may contribute to atherosclerotic calcification [117]. Moreover, COMP or COMP-derived peptidomimetics (CCPep24) can protect endothelial cells from flow-induced inflammatory responses by blocking aberrant integrin α5 activation, thus inhibiting atherosclerosis pathogenesis [118]. COMP binds directly to BMP-2, thus inhibiting BMP-2 receptor binding and blocking BMP-2 osteogenic signaling in VSMC [119] (Figure 6). These findings support the protective role of COMP in mice. However, human study showed that COMP is positively associated with symptomatic carotid atherosclerosis, plaque area, and plaque vulnerability [120]. Serum COMP neoepitope (COMPneo, a fragment generated by COMP degradation) can be utilized as a novel biomarker to identify symptomatic carotid stenosis [121]. 

## 6. COMP and Fibrosis

COMP is a fibrillar collagen assembly regulator [122], which implies its potential involvements in fibrosis (Figure 6). Elevated expression of COMP in skin fibroblasts is associated with systemic sclerosis [10,123] and skin keloid [124,125,126]. COMP levels are significantly increased in the stroma of fibrotic lesions in patients with localized scleroderma [125], and up-regulated COMP stimulates the deposition of collagen and other matrix proteins, leading to additional fibrosis exacerbation [124]. COMP expression levels were higher in larger keloids (>10 cm^2^) than in smaller keloids, implying that COMP expression levels were related to disease progression and might be used as a biomarker to identify disease severity [124]. Indeed, it has been identified as a causal factor promoting collagen deposition in fibrotic skin diseases by modifying fibroblast functions. TGF-β signaling is critical for skin fibrosis [28,127]. Up-regulated by the activation of TGF-β1, COMP stimulates fibroblasts to induce excess matrix deposition [10,123,128] (Figure 6). Recently, Moon et al. discovered that the PI3K-Akt signaling pathway is closely related to skin fibrosis score through gene-set enrichment analysis, and COMP is one of the leading genes of PI3K-Akt pathway in skin fibrogenesis [29] (Figure 6). 

Hepatocytes are the main source of COMP in the liver, and Kupffer cells and hepatic stellate cells also express it but to a much lesser degree [129]. COMP promotes the progression of hepatic fibrosis via CD36/MEK/ERK signaling to facilitate collagen-I deposition in hepatic stellate cells [129] (Figure 6). Serum COMP has been demonstrated to be a sensitive non-invasive diagnostic biomarker for liver fibrosis [122]. Gatselis et al. discovered that serum COMP is efficient to detect cirrhosis in the patients with chronic liver diseases [30]. 

The involvement of COMP is also implied in idiopathic pulmonary fibrosis (IPF). COMP was secreted mainly by mesenchymal cells and most probably fibroblasts in lung [130]. Serum COMP in IPF patients is significantly higher than that in healthy individuals and is correlated with declines in force vital capacity in a time-dependent manner, indicating that it is a potential biomarker for disease activity [130]. COMP expression is enriched in the dense fibrotic regions of the IPF lungs [130], indicating that COMP may play a role in the pathogenesis of IPF. Indeed, TGF-β1 induced PAI1 and COL1A1 can be abolished by COMP silencing in lung fibroblasts [130] (Figure 6).

## 7. COMP and Other Diseases

Serum COMP can be served as a useful biomarker for screening bone and cartilage involvement in psoriasis patients [131] and help distinguish psoriatic arthritis from OA [132,133]. COMP localization of the skin in psoriatic patients extends deeper into the dermis than healthy skin, forming more continuous layers at the nonlesional areas and partially discontinuous deposits at the lesional sites of the dermis-epidermal junction [134]. COMP can interact with α5β1-integrin of basal keratinocytes through the disrupted basement membrane, perhaps stabilizing the epidermis in an atraumatic state by contributing to the suppression of keratinocyte proliferation [134]. COMP may also be a novel modulator of adipose tissue (AT). The levels of COMP mRNA in AT and plasma COMP concentrations were positively correlated with body mass index/obesity [135]. Exogenous COMP protein stimulates adipogenesis of subcutaneous abdominal and gluteal preadipocytes, while the mechanism is still unknown [135]. The D469del-COMP mutant mice show an increase in the adipogenic marker Cebps, possibly contributing to the imbalance between adipogenesis and osteogenesis in mutant mice [15]. Using proteomic analysis, Janša et al. identified that COMP is one of the 16 proteins with different levels in peritoneal fluid of endometriosis patients compared with controls [136]. Although the cells that secrete COMP are unknown, they reported that COMP could be a potential diagnostic and predictive biomarker of endometriosis [136].

## 8. Conclusions and Perspective

With a long-term effort of more than 20 years, notable understandings of the pathophysiology of COMP-related genetic diseases have been achieved, and novel mutations in COMP gene continue to be discovered [51,137]. Protecting chondrocytes from stress is a central mission in the PSACH treatments. Administration of resveratrol in PSACH patients at an early age (approximately 2 years after birth) may be a promising treatment that activates autophagy and clears mutant-COMP retention [138]. Anti-inflammation and anti-MMPs reagents may also be important options to reduce the catabolism effects in the PSACH cartilage. Exercises to increase muscle strength and braces to reinforce loose joints are supplementary for adult patients to delay the onset of OA. Gene therapy remains potential in COMP-related genetic diseases, while it seems out of reach because of tremendous obstacles in this field. 

The potential of serum COMP as a biomarker is well-characterized, at least, in OA. However, the broad expression location and complex connections with multiple diseases impair the specificity of COMP. To date, COMP is a non-specific biomarker, such as erythrocyte sedimentation rate or C-reactive protein, serving as clinical alarm signals. More studies are required to define the gold criteria that can be used for diagnosis and differential diagnosis. Combination with other biomarkers may help to improve the specificity of COMP. Recent studies have implied that tumor expression of COMP can be a good diagnostic and prognostic indicator in human cancers. More importantly, COMP seems to be more relevant to metastasis than malignancy onset, suggesting that it has a critical role in cancer cell behaviors, such as migration and invasion. 

Indeed, accumulating findings have indicated that COMP promotes the aggressive behaviors and stemness of cancer cells. However, the expression of COMP in stroma and cancer cells is rarely distinguished. Moreover, the immune response and immune cell infiltration have not been assessed in the high COMP-expressing tumors. It is not surprising that COMP may modify the interaction between cancer cells and immune cells. As previous studies indicate, COMP is a potential target for cancer, giving promise to the anti-COMP therapy. However, COMP is widely expressed in many kinds of tissues and shows distinct functions in different diseases. Systematic administration of COMP-targeting regent in cancer patients may withdraw its beneficial effects to the vascular system and induce or aggravate cardiovascular diseases. It may be limited to cancer patients without basic cardiovascular diseases. In this case, local targeted therapy is needed. Recent achievements of nanoparticle-based RNA indicate that RNA-based therapy delivered by nanotechnology may be a promising way to go. Since COMP interacts with other proteins to facilitate its functions, targeting the interactions rather than COMP expression may be a promising approach for preventing tumor progression. Due to its protective effects on cartilage and vascular system, COMP supplementation is a potential treatment for OA and cardiovascular diseases. However, potential tissue fibrosis or cancer metastasis needs to be considered because of its notable functions in these diseases. 

## Data Availability

Not applicable.
